# Genome-wide discovery of G-quadruplexes in barley

**DOI:** 10.1038/s41598-021-86838-3

**Published:** 2021-04-12

**Authors:** H. Busra Cagirici, Hikmet Budak, Taner Z. Sen

**Affiliations:** 1 Crop Improvement and Genetics Research Unit, Western Regional Research Center, U.S. Department of Agriculture - Agricultural Research Service, 800 Buchanan St, Albany, CA 94710 USA; 2 Montana BioAg Inc., Missoula, MT USA; 3 Agrogen, LLC., Omaha, NE USA

**Keywords:** Genome informatics, Plant genetics

## Abstract

G-quadruplexes (G4s) are four-stranded nucleic acid structures with closely spaced guanine bases forming square planar G-quartets. Aberrant formation of G4 structures has been associated with genomic instability. However, most plant species are lacking comprehensive studies of G4 motifs. In this study, genome-wide identification of G4 motifs in barley was performed, followed by a comparison of genomic distribution and molecular functions to other monocot species, such as wheat, maize, and rice. Similar to the reports on human and some plants like wheat, G4 motifs peaked around the 5′ untranslated region (5′ UTR), the first coding domain sequence, and the first intron start sites on antisense strands. Our comparative analyses in human, Arabidopsis, maize, rice, and sorghum demonstrated that the peak points could be erroneously merged into a single peak when large window sizes are used. We also showed that the G4 distributions around genic regions are relatively similar in the species studied, except in the case of Arabidopsis. G4 containing genes in monocots showed conserved molecular functions for transcription initiation and hydrolase activity. Additionally, we provided examples of imperfect G4 motifs.

## Introduction

DNA and RNA sequences often form functional secondary structures, such as loops, hairpins, duplexes, triplexes, and quadruplexes^1,2^. G-quadruplexes (G4) are four-stranded nucleic acid structures formed within guanine (G) rich sequences. Consecutive G bases form G-stems (also called G-islands^3^ or G-runs^4^), which make up one strand of a G4 structure. The final form of G4 structures is denoted by numerous G-stems separated by relatively flexible loop regions. Four strands of G bases form square planar G-quartets (reviewed in detail by Kolesnikova and Curtis^4^). The stacking and bonding of these G-quartets then form the final arrangement of the G-quadruplex structures, which is further stabilized by monovalent cations.

G4 structures have been associated with important biological functions including gene expression and regulation^5^, replication^6^, recombination, alternative splicing^7^, and genome stability^8^. In the eukaryotic genomes, G4s are mainly located at the telomeres^9^ and gene regulatory regions such as promoters^10^ and the untranslated regions (UTRs)^11^. An earlier study showed that a ligand-induced G4 stabilization can mediate cell death by the introduction of DNA double strand breaks^12^. In another study, nucleus staining with quadruplex-specific antibodies showed that quadruplex structures were unwound during replication^6^. There is also evidence of the formation of G4 structures at the RNA level where the formation of G4s in the 5′ UTR regions of mRNAs can hinder translation^5,13^.

In plant species, evidence regarding the formation and stability of the G4 structures has steadily been increasing. In vitro studies using circular dichroism spectroscopy showed that formation of stable G4 structures resulted in stalled DNA replication in both monocot and dicot plant species^14^. Further, a recent study showed G4 structure formation *in viv*o and in vitro using chemical structure probing, providing evidence for G4 structures associated with plant growth in both *Arabidopsis thaliana* and rice^15^. Taken together, these experimental studies demonstrated strong evidence of the formation of stable G4 structures as well as the regulatory and functional importance in gene regulation.

In the literature, the nucleotide sequences that have the potential to form G4 structures (G4 motifs) have been primarily defined by the perfect discontinuous G-stems without any mismatches separated by the loops no longer than seven bases. However, experimental evidence revealed exceptions to this rule^16,17^, with the discontinuities in G-stems by mismatches or bulges and with longer loops, expanding our understanding of the G4 structures and leading to alternative predictive models for G4s. For example, Mukundan and Phan confirmed in vitro the formation of imperfect G4 structures containing bulges/mismatches within the G-stems^18^. The first atomic resolution structure of a stable G4 structure, which also possessed mismatched base pairings was published recently^19^. In another study, Palumbo et al. provided an example of a stable G4 structure formed by consecutive G-stems separated by long loops in the hTERT promoter in human^20^. Interestingly, the same study also demonstrated that longer sequence patterns with 12 G-stems has the potential to form higher-order structures of multiple G4s^20^, called multimeric G4 structures. Similar higher-order structures have been reported in other human studies as well, which characterize a crosstalk between adjacent G4 structures to regulate gene expression in the c-KIT promoter^21^. Additionally, these higher order structures might contain imperfect base pairing within the G-stems. For example, Mukundan et al. showed that the HIV-1 integrase inhibitor T30177 forms a multimeric G4 structure through stacking of two consecutive G4 structures containing bulges^22^. Taken together, we now understand that the G4 structures are not always “perfectly” formed but they include G4 structures with (1) imperfect G-stems, (2) longer loops, and (3) multimeric structures. That being said, comparative studies observed the highest stability for the G4 motifs containing at least four G-stems, with at least 3 consecutive G bases, separated by the loops no longer than 7 bases, and the lowest stability in the imperfect G4s^23,24^. Therefore, computational studies largely focus on the most stable, perfectly defined G4 motifs, with some cases of imperfect G4s, a convention we also follow here.

Identification and characterization of regulatory elements is a prerequisite for fully understanding the transcriptional landscape of a genome, which in turn facilitates developing new approaches for crop improvement. Although reference sequences have been sequenced, assembled, and released for many plant genomes^25,26^, only a limited number of genome-wide studies have been performed to identify G4 structures in plants. In this study, putative G4 motifs were identified in barley (*Hordeum vulgare*) using bioinformatics methods, and a detailed investigation of the genome-wide distribution of G4 motifs was performed with respect to regulatory elements. As we explained above, since the imperfections in the G4 motifs resulted in the decreased stability^2,25^, we put our main effort on the identification and analysis of the standard G4 motifs. Our results showed that G4 motifs in the barley genome were enriched at the 5′ UTR, the first CDS, and intron 1 start sites on antisense strands. Our study also provides case studies of imperfect G4 motifs containing mismatches within the G-stem and multimeric G4s that contain at least eight G-stems. We also compared the G4 motifs in barley with wheat and other monocot plants. Functional annotation of the genes containing G4 motifs revealed several molecular functions conserved between among phylogenetic taxa. Additionally, single nucleotide polymorphisms (SNPs) that might affect the stability and the formation of the quadruplex structures were identified for further studies.

## Results and discussion

### Genome-wide G4 motifs in barley

Genome-wide studies have showed the formation of stable G4 structures in plants^27^ with some studies revealing that G4 motifs are more abundant in monocots than in dicots^14^. Here, we identified G4 motifs in the barley genome using (G_3+_L_1−7_)_3+_G_3+_ as the perfect search pattern. This pattern identified G4 motifs that are constructed of at least four runs of G-stems separated by loops of one to seven nucleotides^24^, forming at least three G-quartets. In total, 329,098 G4 motifs were identified in barley, showing a G4 density of 77 motifs per Mb. The sequences and genomic locations of putative G4 motifs in barley were provided in Supplementary Data 1 and are available at the GrainGenes repository (https://wheat.pw.usda.gov)^28^. The observed density was much higher than that found within the previously reported dicot plant, *Arabidopsis thaliana* (G4 density of 9 G4 motifs per Mb)^29^. (To prevent any bias introduced by different identification protocols, we re-identified G4 motifs in the Arabidopsis genome in accordance with the methods used by previous reports^29^ and observed a similar G4 density of 10 G4s per Mb). Our results also showed that, similarly to what was observed in barley, G4 density was higher in the other monocot plants analyzed (maize, wheat, and rice) (Supplementary Table 1) than in the dicot plant, Arabidopsis.

The literature shows G4 enrichment in genic regions around specific regions such as promoter, UTR, and CDS regions in human, Arabidopsis, rice, and wheat^30–32^. To investigate the distribution of G4 motifs with respect to key gene structural regions in barley, annotations for high-confidence (HC) genes were downloaded and the prevalence of G4 motifs across genic regions was analyzed. Overall, ~ 1.2-fold increase to 93 G4s per Mb in the G4 motif density was found around genic regions when compared against the whole genome (Supplementary Table 1). To evaluate the enrichment of G4 motifs within genic regions, we compared the G4 density in the high-confidence genes with that obtained from randomly shuffled sequences across the genome (See “Methods” section). We observed 1.5-fold higher G4 density in genic regions against the random genome-wide shuffling, which means that high-confidence genes in barley contain more G4 motifs than would be expected randomly.

**Figure 1:**
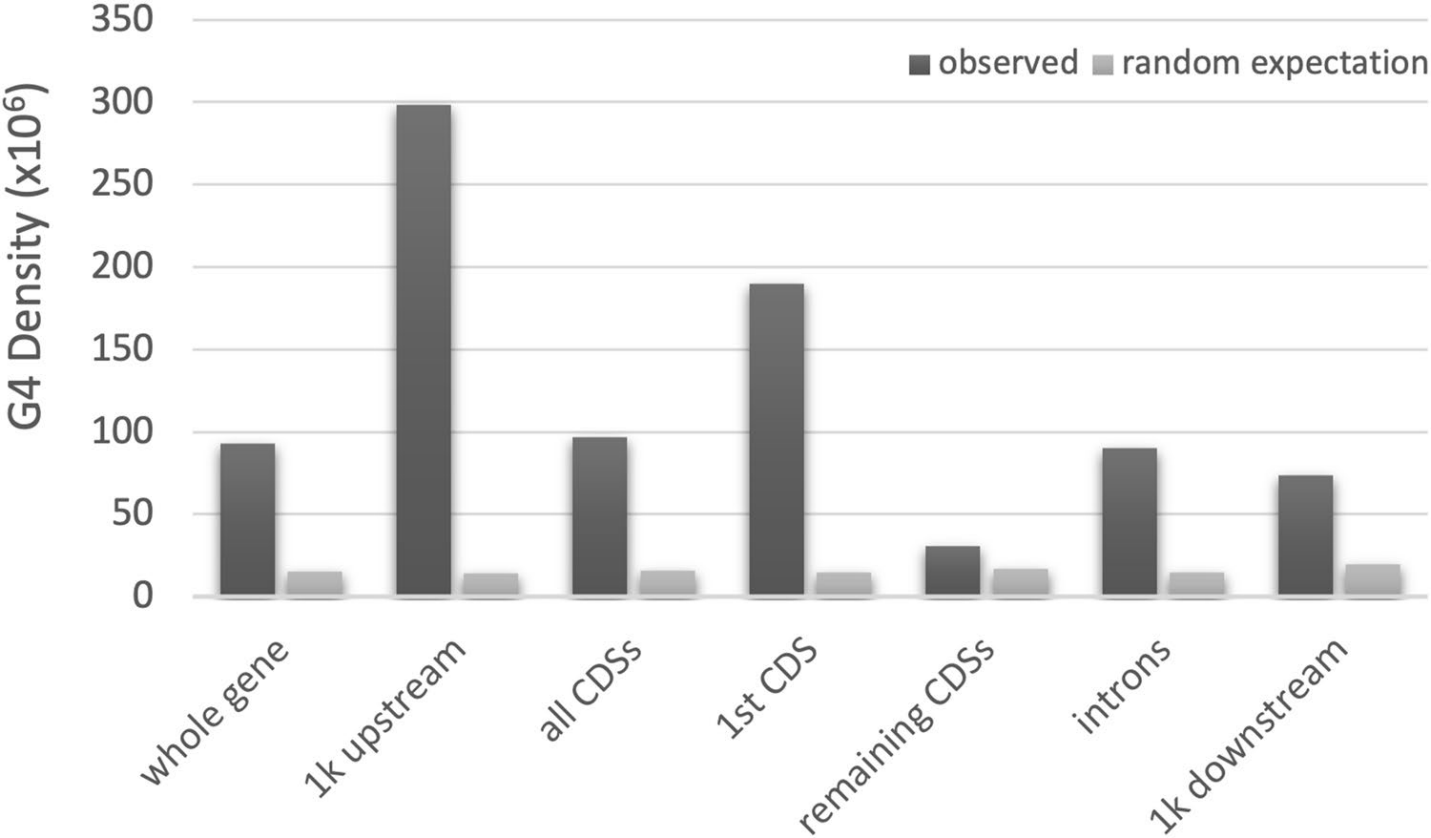
G4 density distribution across gene structural elements. G4 density was calculated as the total number of G4 motifs overlapping with each target region, per Mb. Target regions include 1k (1000 bp) upstream of the gene start codon, 1k downstream, CDSs, introns, and the whole genes.

Further, we investigated the G4 motif densities in each gene structural elements (Fig. 1). The G4 density plot shows that G4 motifs are the most prominent in the 1 kb upstream of the gene start codon. Within the whole gene, the densities between CDSs and introns were very similar; however, G4s were distributed unevenly within CDSs. More than 80% of the G4s in the CDSs are located in the first CDSs, resulting in ~ 6 times higher G4 density in the first CDSs than the remaining CDSs (Fig. 1). Comparison to the randomly generated sequences revealed that observed densities were larger than the densities in the random data (Fig. 1).

**Figure 2:**
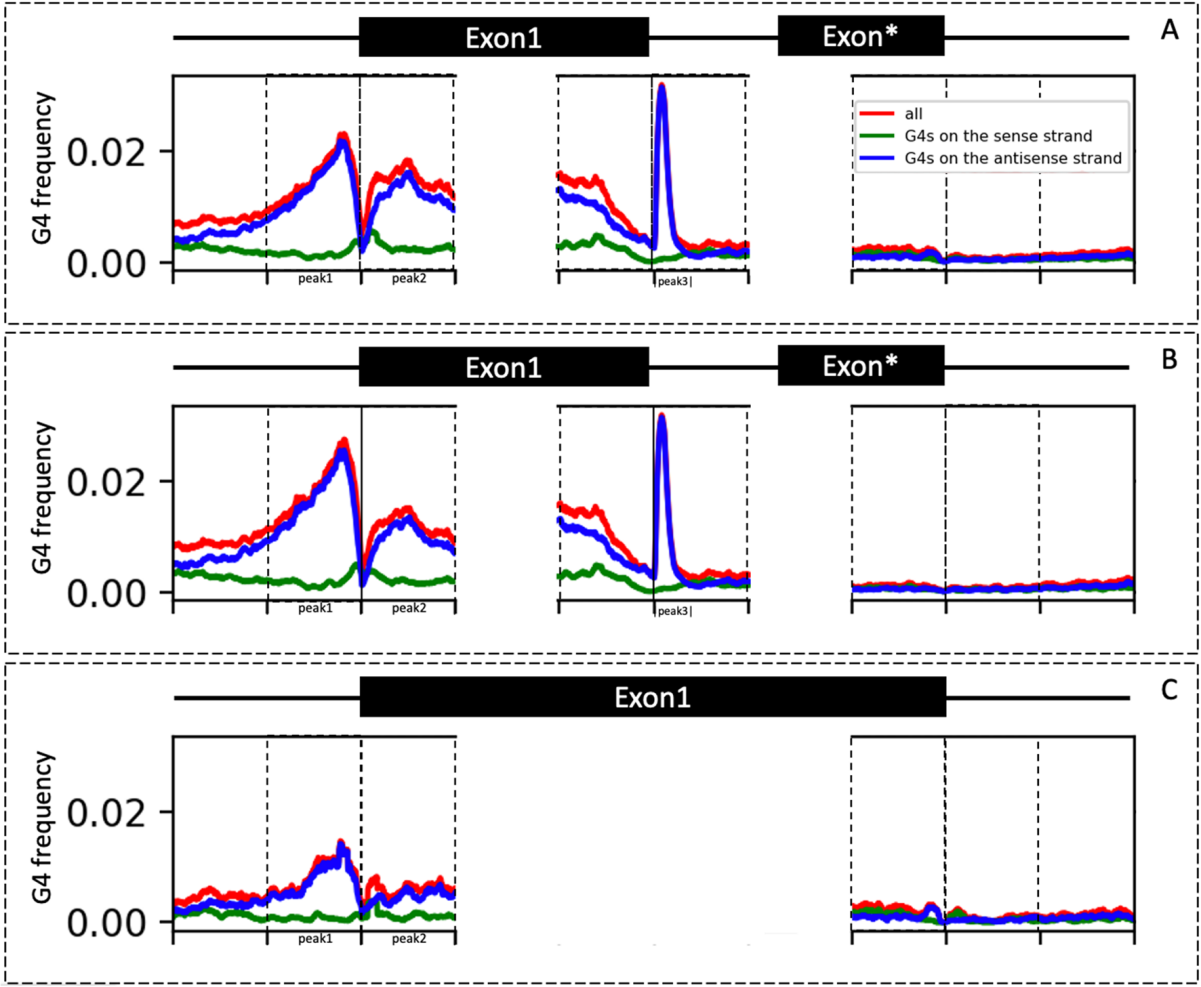
G4 motif frequency in a barley gene. G4 motifs on the sense strand (green), G4 motifs on the antisense strand (blue), and G4 motifs on both strands combined (red) are shown. Exons are shown by black boxes connected with introns (black line). Exon* represents the last exon of a high-confidence gene. Graphs show the frequency of G4 motifs at each position without any smoothing. G4 motifs overlapping genes and 500 bp flanking regions were calculated for all high-confidence genes. Equally sized 250 bp regions were separated by dashed lines. G4 frequency was calculated for (**A**) both multi exon and single exon gene models (a total of 32,159 sequences), (**B**) multi-exon gene models (22,171), and (**C**) single-exon gene models (9988). G4 frequency was calculated by dividing total number of G4 motifs overlapped each position by the total number of regions for each position.

Later, we examined the enrichment of G4 motifs within the 10 kb region centered at the first CDS start site (Supplementary Figure S1). Consistent with previous studies^30–32^, we observed the enrichment of G4 motifs over the 1000 bp region centered at the CDS start site (Supplementary Figure 1 and Figure 2), covering the 5′ UTR and the first 500 bp of the first CDS. Since the most up-to-date annotation data for barley does not contain the UTR information, we did not separate the 5′ UTR and promoter regions. Instead, we arbitrarily defined the 500 bp upstream and 500 bp downstream of a gene as gene regulatory regions of 5′ UTR and 3′ UTR, respectively. Including the 500 bp flanking regions corresponding to UTRs, the total number of G4 motifs within the genic regions increased from 9360 (Supplementary Table 1) to 16,265. Interestingly, more than 60% of the G4 motifs overlapping with the genes and UTR regions were located within the1000 bp region centered at the gene start codon (Supplementary Figure 1 and Figure 2). Together with previous reports on G4 motif distribution in other phylogenetic taxa^29,31,33^, these results imply that there might be minor differences in the distribution of G4 motifs over genic regions among different species. Assuming that the promoter region resides in the 1 kb upstream of the start codon, the enrichment of G4 motifs within close proximity of promoter and TSSs appear conserved across many species, thus supporting the hypothesis linking the role of G-quadruplexes to gene transcription and translation regulations^34,35^.

A closer examination of the G4 motif distribution with respect to gene structural elements provided interesting observations. Positional G4 frequency distribution (Fig. 2B) shows the highest enrichment on the antisense strand of the genes at the intron 1 start site, within a 100 bp region called peak3 for the genes with multiple exons. The peak3 region is the exon–intron boundary, which contain specific splicing signals like polypyrimidine tracts^36^, where some studies reported the presence of multiple G-triplets^37^. Indeed, several studies showed G4 structure formation in the intron splice sites on the antisense strand^7,36^. Here, our results showed an enrichment of G4 motifs, with the most striking difference at the intron 1 start site, further suggesting that G4 motifs may be playing a functional role in guiding the mRNA splicing in barley. Our results also showed two peaks around the gene start codon (Fig. 2). Peak1 was found 250 bp upstream of the gene start codon (5′ UTR; -250 bp to start codon) and peak2 was the first 250 bp of the gene start (from the start codon to + 250 bp). Similar to what has been reported in previous studies on human^38^ and some plant genomes, including wheat^31^, G4-enriched regions are depleted of G4 motifs at the CDS start site in barley. G4 frequency showed variation between single- and multiple- exon genes (Fig. 2B and C), with multiple exon genes having considerably higher enrichment at peak1. G4 frequency distribution also showed depletion of G4 motifs within the last exon and the following 500 bp region, spanning the 3′ UTR region.

To determine chromosome specific and/or strand specific characteristics, the distributions of G4 motifs across all chromosomes in both strands were investigated. Enrichment of G4 motifs around CDS start sites was observed across all chromosomes with a higher prevalence on antisense strands (Supplementary Figure 1). This is in agreement with the previous reports on the distribution of G4 motifs in the monocot plants where antisense strands are favored^31,33,39^. The human genome, on the other hand, showed enrichment of G4 motifs around TSSs on both sense and antisense strands^38,40^ (with some studies^41^ showing that G-rich regions are biased in the antisense strand). The deployment of G4 motifs on the sense strands in monocots plants may suggest a divergent evolutionary pressure on the G4 motifs for biological functions in different species. On the other hand, the abundance of the G4 motifs around genes either in the sense or antisense strands in different phylogenetic taxa could reflect regulatory functions independent of the strand such as a nucleation factor for a fast folding process^42^ or recruitment of the chromatin remodeling complexes and histone chaperones^43^, or the molecules involved in DNA repair, helicases, or translation^44^.

### The effect of smoothening due to window size

**Figure 3:**
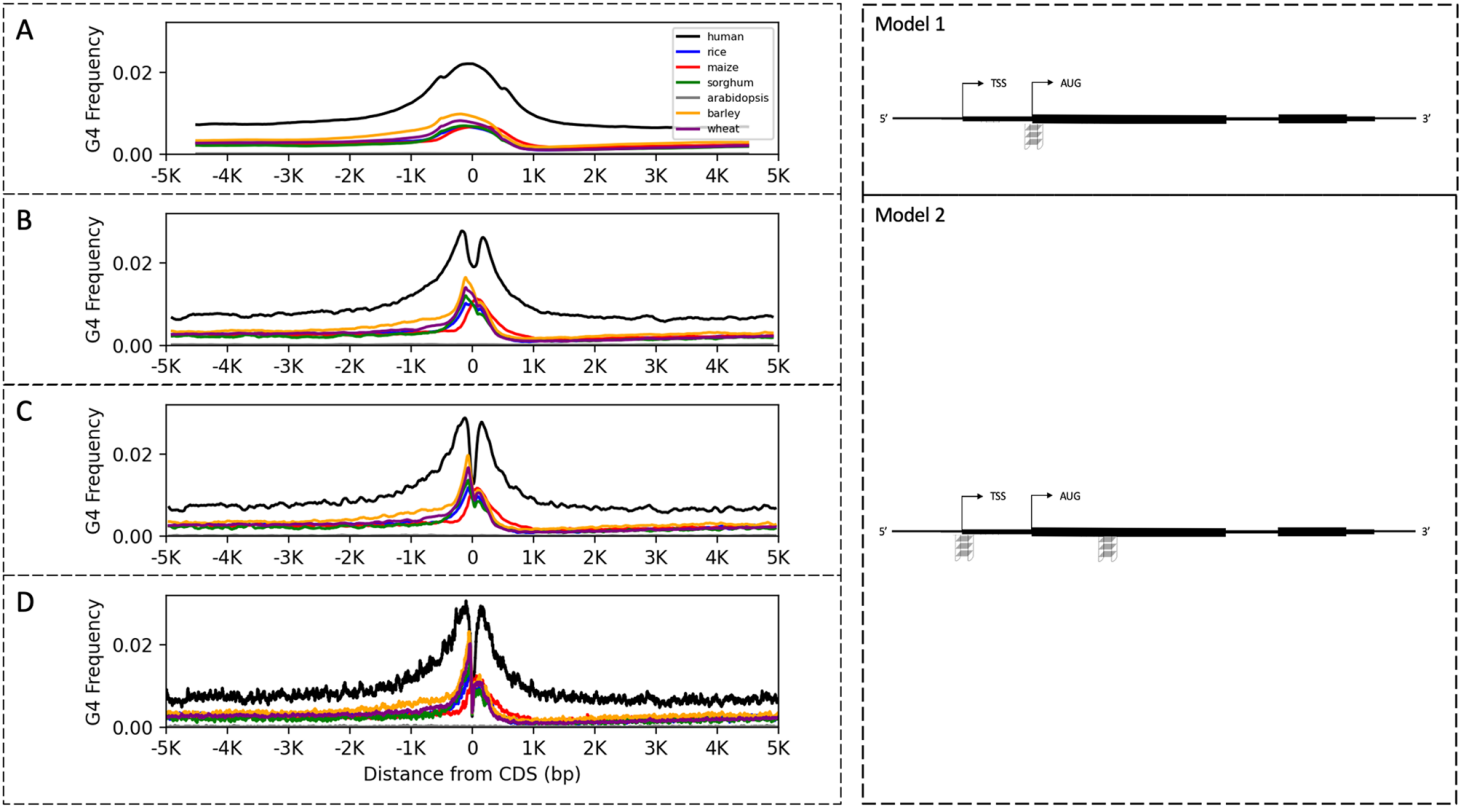
G4 motif frequency per Mb over 10,000 bp region centered at the first CDS start site. Moving averages were calculated with a window size of (**A**) 999 bp, (**B**) 199 bp, (**C**) 99 bp, and (**D**) 1 bp. Genomes colored as black for human, blue for rice, red for maize, green for sorghum, gray for Arabidopsis, orange for barley, and purple for wheat. On the right, predicted G4 motif distributions over genic regions are shown based on the corresponding abundance profiles of the plots on the left. Arrows illustrated the direction of transcription. CDS regions are shown as black boxes, UTR regions as the flanking regions from the gene ends, and the introns as the regions connecting CDSs. Model 1 is a prediction based on the G4 frequency graph averaged out with a window size of 1000 bp with a single peak and Model 2 is with a window size of 100 bp with a double peak. Inverted G4 structures indicate motifs on the antisense strand.

Many studies represented the G4 motif distribution by taking averages within large sliding windows^33,45^. It is important to note that the use of moving averages with larger window sizes may conceal peak points. Although these approaches are useful to give a broader view of G4 distribution, they may also hinder the understanding of more specific G4 populations within the genome. For example, Wang et al. showed the distribution of G4 motifs in the rice genome within a window size of 200 bp^33^ and Takahashi et al. showed G4 motif distribution in human, rice, and Arabidopsis genomes using a window size of 1000 bp^45^. Those studies did not mention the two peaks and the sudden decrease in the G4 density at the start codon or TSS. Here, we compared the effect of window sizes. Figure 3 illustrates the smoothening effect of the differently sized windows. We plotted the G4 frequency distribution centered at the start codon rather than 5′ UTR start site for every genome investigated. With a window size of 1000 bp, only a single peak of G4 enrichment was observed (Fig. 3A). In contrast, with a window size of 200 bp, two enrichment peaks were clearly observed in human, though not as discernable in plants (Fig. 3B).

With larger window sizes to average out the G4 density over genic regions, G4 distribution prediction appeared as a large single peak around the start codon (Fig. 3-Model 1). However, with no smoothening or with small window sizes, two peaks emerge as hotspots on the antisense strand located near TSSs and the first CDS (Fig. 3-Model 2).

Our results showed that a window size of 100 bp showed both a smooth and precise distribution of G4 density in both mammalian and plant genomes, and future studies should be cautious of the smoothing effect of larger sliding windows for their species (Fig. 3C).

### The effect of the availability of 5′ UTR region information

We also observed that the distribution of G4 motifs over gene start sites was highly dependent on the availability of gene annotations. Although the knowledge of 5′ UTR regions provide highly valuable information, they are not always available in gene annotations, which contributes in part to the inexact calculation of G4 distribution in gene structural elements. Even the latest genome annotations of most plant genomes^26^ have many genes without associated 5′ UTR information. Therefore, a possible source for discrepancies in G4 motif distribution calculations may be the incompleteness of annotation data for 5′ UTR regions.

### Imperfect G4 motifs

**Figure 4:**
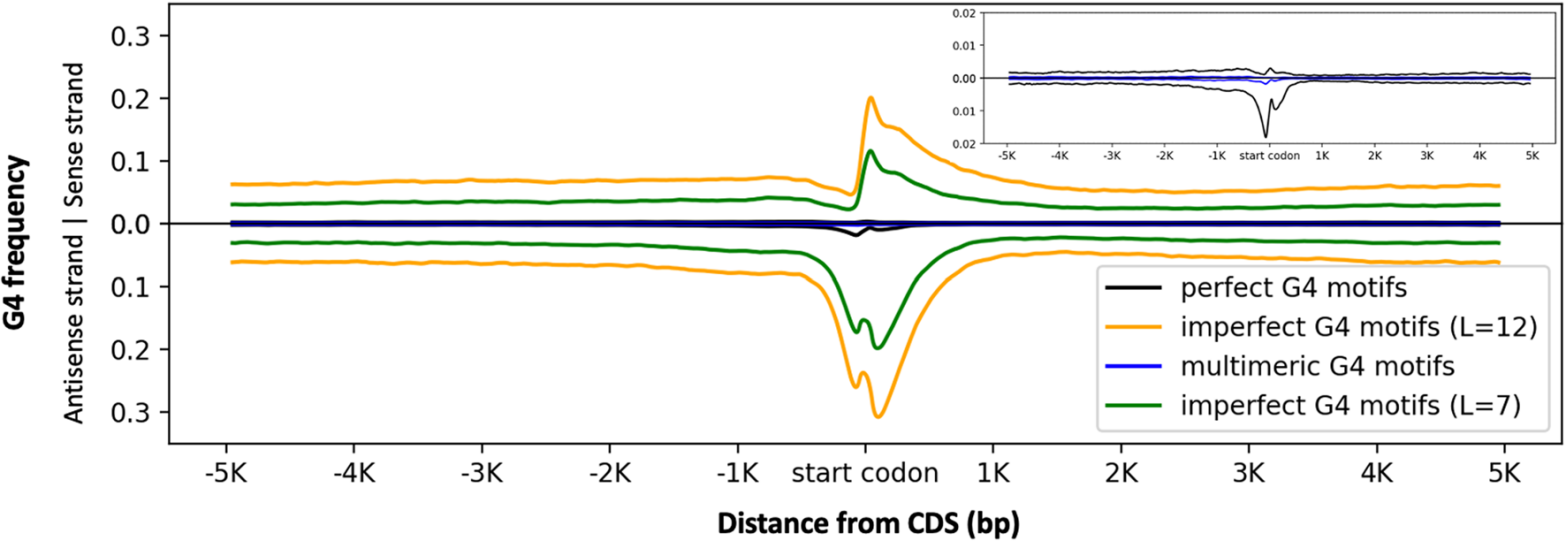
G4 frequency distribution of different G4 motifs over the 10 kb region centered at the high-confidence gene start site. X-axis represents the location with respect to start codon. Y-axis represents the G4 frequency which was calculated by the average number of G4 motifs at each position per Mb. Represented motifs are perfect G4 motifs (black), multimeric perfect motifs (blue), imperfect G4 motifs (green), and imperfect G4 motifs with long loops (orange). A focused plot on the top right corner shows the G4 motif distribution for the G4 frequency smaller than 0.02.

To identify the imperfect (or degenerate) G4 structures with discontinuous G-stems, we used the QPARSE algorithm^46^. We specifically examined imperfect G4 motifs that are defined as sequences with loops up to 7 bases, with G-stems of length 3 or 4 bases, with maximum one bulge and the length of the bulge up to 2 bases. Overall, total number of imperfect G4 motifs (5,884,180) were ~ 18 times higher than the perfect G4 motifs. In general, perfect and imperfect G4 motifs showed similar distributions, where ~ 5% of the both perfect and imperfect G4 motifs were located within 500 bp flanking regions of the high-confidence barley genes. The enrichment patterns were varied between imperfect and perfect G4 motifs: imperfect G4 motifs peaked at (1) upstream and (2) downstream of the start codon on the antisense strand, and (3) the downstream of the start codon on the sense strand (Fig. 4).

As a case study to examine imperfect G4 motifs, we ran the QPARSE algorithm using the same parameters as before for the imperfect G4s, except increasing the loop length to 12 (-L 12). Total number of imperfect G4 motifs is almost doubled to 9,419,305 by increasing the maximum loop length from 7 bases to 12. G4 frequency distribution over the 10 kb centered at the start codon revealed a similar pattern for imperfect G4 motifs (L = 7) but differed in magnitude (Fig. 4). Previous studies demonstrated examples of imperfect G4 motifs with long loops that played conflicting roles in G4 stability by either destabilization^47^ or stabilization^20^ of G4 structures. For example, Guedin et al. reported that increasing the loop length leads to a decrease in stability^47^; although, other studies reported stable formation of G4 structures with long loops^48,49^. Therefore, the ability of predicted imperfect G4s forming stable structures requires further experimental support.

### Higher-order structures

Given the evidence of multimeric G4 structures in the promoter regions of the human genes^20,21^, we analyzed the distribution of the G4 motifs that have the potential of forming multimeric structures in barley. We searched for the putative multimeric G4s across the barley genome which we defined here as the perfect motifs containing at least 8 putative G-stems ({G_3+_L_1−7_}_7+_G_3+_). It is important to note the imprecision of predicting dimeric and trimeric G4s computationally: among the defined G-stems within the computationally predicted G4 motifs, some might participate in the base pairing to form G-tetrads whereas some might take part in the loop regions and called as loop instead of G-stem. In our study, we disregarded these cases and identified 13,780 putative multimeric motifs that have the potential to form multimeric structures with at least 8 G-stems across the barley genome. 594 (1.85%) of the high-confidence barley genes contain at least one multimeric G4 motif within the gene body and 500 bp flanking regions. Multimeric G4 motifs were the most abundant at the upstream of the start codon on the antisense strand (Fig. 4). We also observed depletion of multimeric G4 motifs on the sense strand at the gene start codon.

### G4 motif distribution across barley and wheat genomes

Both barley and wheat have undergone artificial selection and divergence, as both species have been grown for food production since the agricultural revolution ~ 10–11 thousand years ago^50^. Wheat differs from barley in that it is an allohexaploid, which resulted from hybridization of three species, each containing seven pairs of chromosomes for a total of 21 pairs. In comparison, barley is diploid with only seven pairs of chromosomes. As close relatives, most of the chromosomes exhibited high degree of similarity between the two genomes^51^. Indeed, further studies supported the conservation in gene content and order with a few wheat-specific rearrangements leading to local reordering in the wheat genome^52,53^. Our initial results demonstrated that overall G4 densities across the whole genome and genic regions are similar in the barley and wheat (Supplementary Table 1).

**Figure 5:**
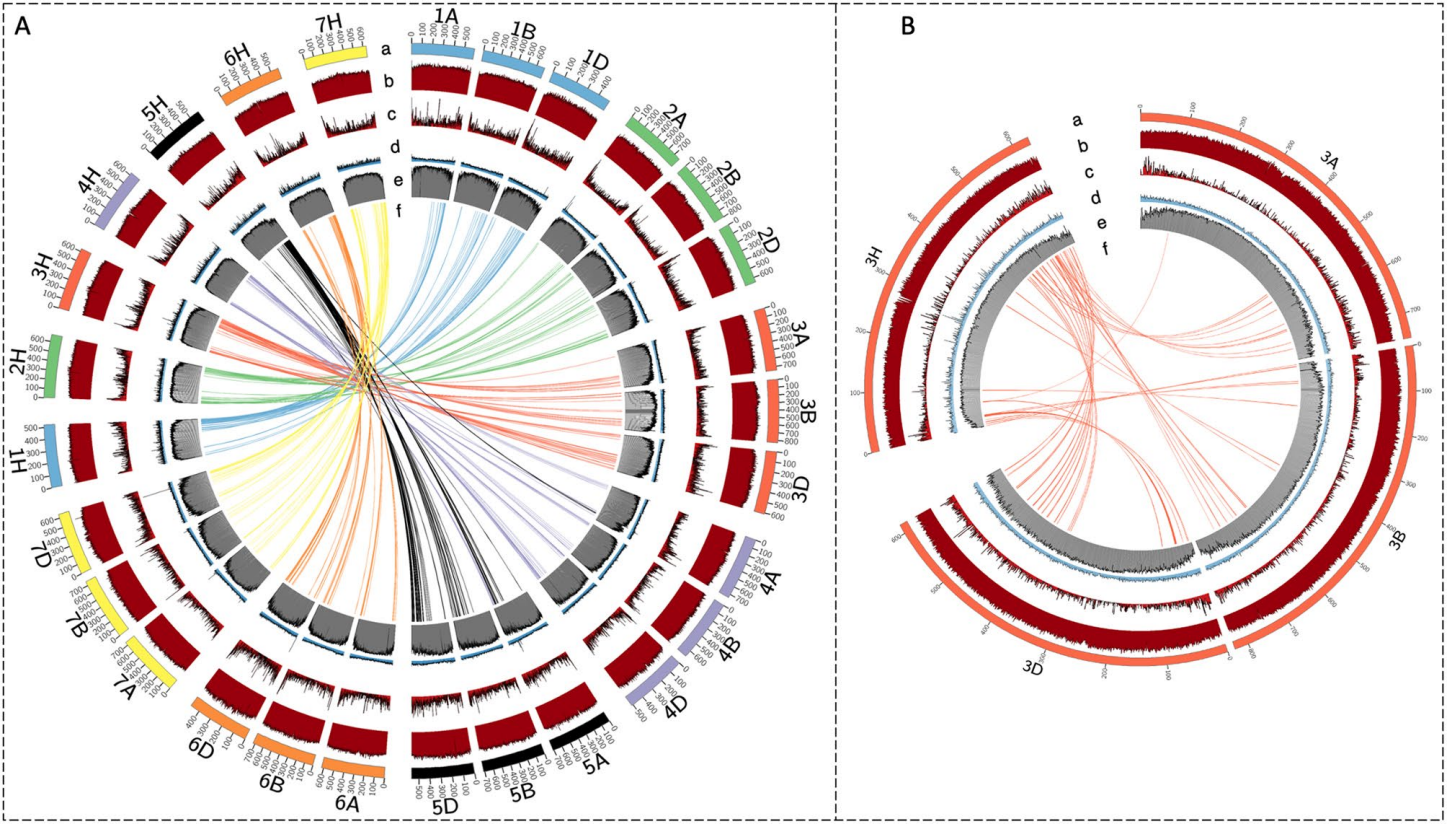
Circos diagram depicting the distribution of G4 motifs together with other regulatory elements in the barley and wheat genomes. Barley chromosomes are the H chromosomes and wheat chromosomes are the A, B, and D chromosomes. The plot shows the regulatory elements as from outside to inside: (a) ideogram the chromosomes (in Mb), (b) line graph of GC distribution as percentage to total number of bases, (c) histogram of high-confidence gene density, (d) line graph of G4 motif frequency, (e) histogram of transposable element density, and (f) syntenic genes between the barley and the wheat genomes. All chromosomes were shown (**A**) in the figure on the left and (**B**) a focused plot of chromosome 3 was shown on the right.

To compare G4 motif distribution across the wheat and barley genomes with respect to HC genes, transposable elements (TEs), and GC content, we created Circos plots^54^ dividing each chromosome into 1000 bins. Figure 5 shows G4 motif distribution together with other regulatory elements in the outer layers and the G4 motif containing genes conserved between wheat and barley in the middle as bundles. Input files used to generate Circos plots are provided in Supplementary Data 3.

Although G4 motifs were reported to be enriched around telomeric regions in humans and Arabidopsis^55–57^, we observed that G4 motifs in barley were distributed throughout the chromosomes (Fig. 5). Our results showed that the G4 peaks observed in wheat chromosomes were not always conserved in barley (Fig. 5A). For example, no complements were found in barley for the peaks at the end of the chromosomes 5A and 7D or the peaks at the start of the chromosomes 2A and 6D in wheat. Yet, a noticeable peak at the distal end of the barley chromosome 4H (at ~ 600 Mb) was also detected at the ~ 500–600 Mb regions on the wheat chromosome 4A. Some peaks were unique to barley such as on the chromosome 3H (Fig. 5B) at the ~ 270 Mb which was annotated as centromere^26^.

We investigated the conservation of the genes containing G4 motifs among the wheat and barley genomes. 4898 homologous genes between barley and wheat were identified using the best reciprocal hits approach (BRH)^58^. Individual syntenic links were bundled so that single links in close proximity merge into a single synteny block (min 3 links in a bundle with max_gap_1 of 1E6) in Fig. 5. Although wheat chromosomes are highly similar between its three subgenomes (A, B, and D), differences between the number of synteny blocks were noticeable. Synteny blocks between the barley and wheat chromosomes for genes containing G4 motifs showed the least similarity between barley and wheat subgenome A, with the exception of chromosome 7A. The highest similarity for G4-containing synteny blocks between the barley and wheat genomes was observed for the D subgenome for all seven chromosomes. Overall, the largest number of homologous genes were located in chromosome 5.

### Repetitive content of the G4 motifs

Transposable elements (TEs) are abundant in eukaryotic genomes, representing up to 90% of the plant genomes^59^. Overall, TEs constitute 80.8% of the barley genome^60^. Our results showed that 211,971 (64.41%) of the genome-wide G4s reside within TEs. Among plant TEs, long terminal repeat (LTR) retrotransposons are the most common in plant genomes, while animal genomes are often dominated by non-LTR retrotransposons. In barley, we found that 85.46% of the TEs overlapping with the G4 motifs were indeed LTR retrotransposons, with the unclassified LTR retrotransposons (RLX) being the most abundant. Gypsy and Copia superfamily of LTR retrotransposons constituted 22% and 20% of the repeats overlapping with the G4 motifs. Other than LTR retrotransposons, CACTA superfamily of the DNA transposons were represented at 13% of the repeats overlapping with G4 motifs. The repeat families overlapping with the G4 motifs are provided, along with their relative abundance, in Supplementary Table 2.

TEs have been shown to be responsible for the evolution of the gene regulation and for chromosomal arrangements^61^. Even the variations in the genome size have been attributed to transposable element content of the genomes^62^. As with the observation that most of the TEs (85%) reside within the G4 motifs in barley, our results suggest that the composition of transposable elements in a genome might drive the genomic spread of the G4s. Together with the previous reports on the enrichment of G4 motifs within the plant retrotransposons^59^, the lack of G4 peaks and the low number of G4 motifs observed in the Arabidopsis genome might be explained by the repetitive content of the genome, where TEs are less abundant in the Arabidopsis genome^63^.

### Functional annotation of the putative G4 motifs

Formation of G4 structures within gene body and/or gene regulatory regions like promoters were associated with the regulation of fundamental biological processes^64^. Studies showed inhibitory functions of G4s^65^ located within gene body or its flanking regulatory regions as well as enhancer functions through recruiting regulatory proteins to a target site^66^. To explore the presence of G4s in these regulatorily important regions, we selected genes containing G4 motifs within their gene body or 500 bp flanking regions and analyzed for gene ontology (GO) term assignments to understand what gene functions may be regulated by G4s. Of 32,159 high-confidence gene models, 10,120 (31.47%) contain at least one G4 motif within the gene body or its flanking regions. All the genes containing G4 motifs, except one, were in fact assigned at least one GO term. Most of these genes were involved in metabolic (56.9%) and cellular (53%) processes in the Biological Process category, and binding (52.7%) and catalytic activities (52.2%) in the Molecular Function category (Supplementary Data 2).

**Figure 6:**
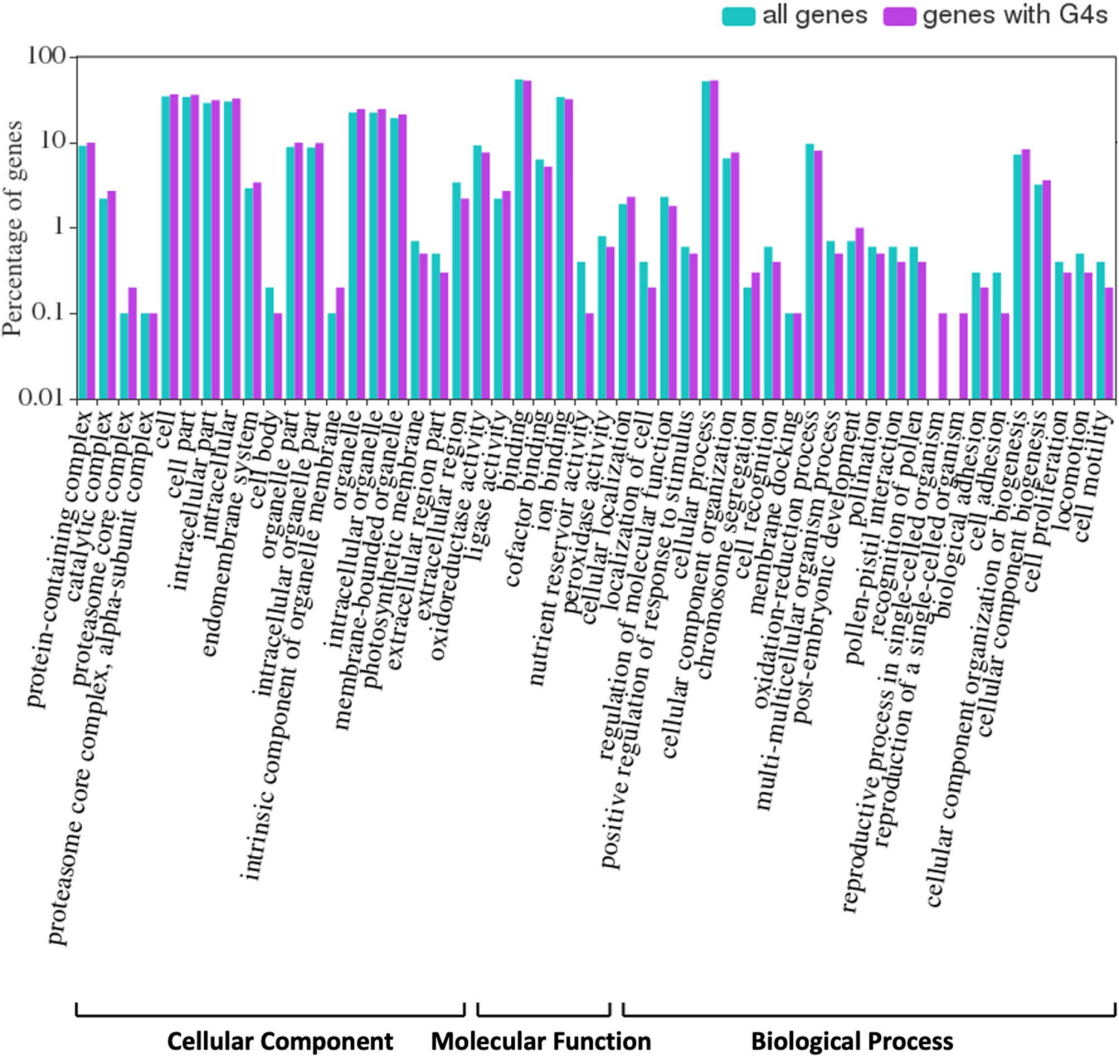
GO terms with a significant relationship between barley high-confidence genes (“all genes”) and the genes containing G4 motifs (“genes with G4s”). Percentage of genes are represented on the logarithmic scale.

A Pearson chi-square test was applied to identify GO terms with significant relationships (p-value < 0.05) in the genes containing G4 motifs against all high-confidence genes. GO terms with a significant relationship were plotted in Fig. 6 for the Cellular Component, Molecular Function, and Biological Process categories at level 2 in the GO hierarchy. Most of the GO terms showed similar distributions between all high-confidence genes and the genes containing G4 motifs; however, reproduction of and reproductive process in single cell organism biological processes were the most linked to genes containing G4 motifs (Fig. 6).

A GO term enrichment analysis was performed for the 10,120 genes containing G4 motifs within the gene body and 500 bp flanking regions, using the BiNGO plugin for the Cytoscape visualization tool^67^. The GO enrichment analysis revealed that G4 motif-containing genes are enriched in 19 GO terms in biological processes, such as in response to cold (GO:0009409), transcription initiation (GO:0006352), and post-embryonic development (GO:0009791). In addition, 9 GO terms in the molecular function category, which are mostly associated with either binding or catalytic activity, are enriched in the G4 motif-containing genes, such as hydrolase activity (GO:0016818 and GO:0016817). All sets of enriched GO terms were provided in the Supplementary Data 2.

Given the evidence of functional similarities among plants, the associations of G4 motifs with these specific functional annotations were also investigated. Among the barley high-confidence gene models, a total of 1192 genes were assigned to hydrolase activity with the GO term GO:0016818. 447 (37%) of these hydrolase genes contained G4 motifs. Overall, the abundance of the genes that were assigned to hydrolase activity over whole barley high-confidence genes was slightly lower than the abundance of these genes over the genes containing G4 motifs. More specifically, 3.71% of the high-confidence genes were annotated with hydrolase activity as opposed to 4.42% of the G4 motif containing genes. Similarly, hydrolase activity (GO:0016818) has been reported in different plant studies^31,45^ as enriched in G4 containing genes.

Cold response is another important biological process for plant survival. The results showed that most genes assigned to response to cold (GO:0009409) function contain G4 motifs. 29 of the 10,120 genes containing G4 motifs were linked to cold response, as opposed to only 21 non-G4 motif containing genes. The conservation of these genes between barley and wheat was also investigated. Sequence similarity between barley and wheat genes based on a best reciprocal hits approach revealed that 27 of these genes are homologous to wheat genes containing G4 motifs (Supplementary Table 3).

Of the 10,120 genes containing G4 motifs within the gene body and 500 bp flanking regions, 6116 (60%) of the genes contain G4 motifs at the peaks on the antisense strand, 3369 genes were located within peak1, 2072 genes within peak2, and 675 genes within peak3 (for peak locations, please see Fig. 2A). GO term enrichment analysis revealed that genes containing G4s within peak2 were not enriched for specific GO terms in the biological process and the molecular function categories when compared to all HC barley genes. On the other hand, genes containing G4 motifs within the peak1 were enriched in 9 GO terms in the molecular function category and 31 GO terms in the biological process category, which include regulation of gene expression (GO:0010468) and regulation of transcription (GO:0006355 and GO:0045449). Genes containing G4 motifs within peak3 were enriched in 7 GO terms in molecular function category. Full list of enriched GO terms and the enrichment results are provided in Supplementary Table 4. Intriguingly, two genes containing G4 motifs within peak3 were the only barley genes that were annotated with 5-oxopent-3-ene-1,2,5-tricarboxylate decarboxylase activity (GO:0018800). These results suggest an association of G4 motifs with unique biological processes depends on the localization in the barley genome.

### Presence/absence of variations within the G4 motifs

Due to the required specificity in forming stable G4 structures, G4 structures can lose their integrity by single nucleotide mutations^68^. Some of these variations might alter the G4 motif, resulting in a change in structural conformation, and therefore impart instability to G4 structures^34,69^. In the human genome, it was shown that G4 motifs, specifically the disruptive positions of G-stems, displayed significantly lower level of small variations than their neutral counterparts^70^, suggesting functional significance.

To explore this in barley, we analyzed disruptive mutations in G4 motifs across the barley genome with a particular focus on their distribution in the vicinity of TSS and genic regions. All G4 motifs distributed over the barley genome were screened for the presence of single nucleotide polymorphisms (SNPs) that were made available through Barley 50k iSelect SNP array^71^. Consistent with the previous reports in human studies^70^, we observed that SNPs were less abundant in the G4 motifs: only 19 SNPs were located within the G4 motifs. We further examined the abundance of G4 motifs containing a SNP position across the gene features and 500 bp flanking regions. As expected from an exome-capture sequencing data targeting only the exome regions, most of the SNPs observed in G4 motifs were located within the genic regions. 16 of these SNP containing G4 motifs, which corresponds to 84% of the SNP containing G4 motifs, overlapped with genic regions.

**Figure 7:**
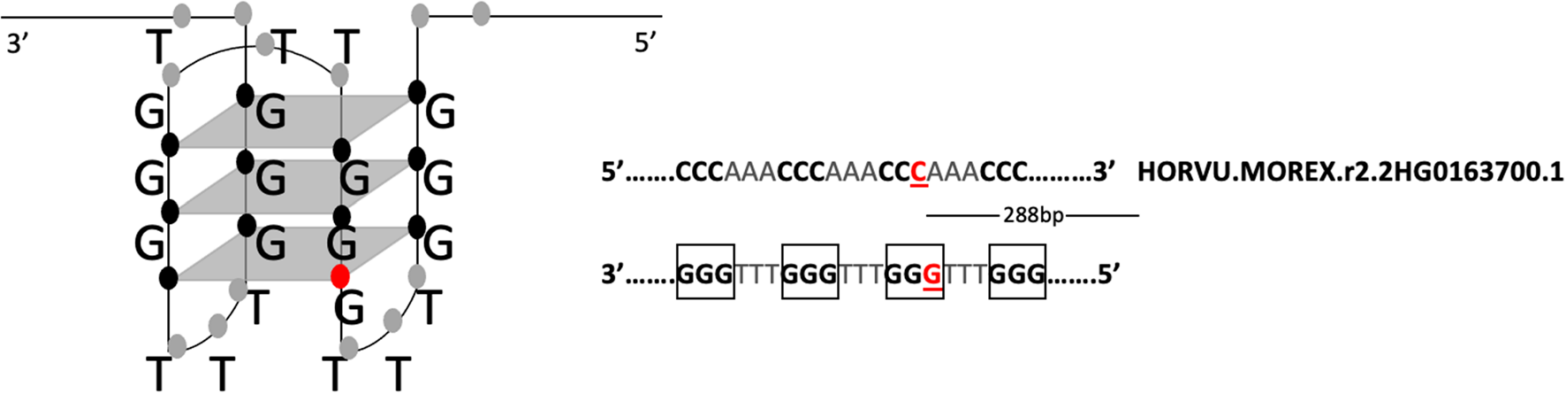
A representation of the localization of SNPs disruptive to G4 structure formation. On the left, the G4 structure is shown with a variation. Red dot indicates changes to the conformation of the bottom G-quartet, black dots indicate possible sites prone to conformation change, and gray dots indicate sites with less influence on G4 structure formation. On the right, the same motif sequence is shown on a linear plane on the sense and the antisense strands. G-stems were indicated as bold within the box. Mutation site is underlined. The distance of the variation to the closest gene is indicated.

Although the formation of stable G4 structures was observed for some G4s containing mismatches and bulges within the G-stem in human studies^22,48^, comprehensive comparative studies suggested that those G4s containing mismatches within the G-stem were less abundant and most of the G4s forming stable structures were indeed comprised of the perfect G-stems without any mismatches^23^. Therefore, we investigated the localization of the SNPs across the G4 motifs with the ability to interrupt the formation of G-quartets. Notably, three of the SNPs were located within the four main G-stems that are required for the proper folding of G4 structures and therefore might directly affect stability of the quadruplex motif. As an example, the JHI-Hv50k-2016-120665 SNP, located upstream of the HORVU.MOREX.r2.2HG0163700.1 gene is shown in Fig. 7. This gene was annotated as a PLATZ transcription factor. Intriguingly, this PLATZ gene is known to play significant roles in regulating plant development and stress resistance, and has been shown to exhibit tissue and stress specific expression in plants^72^. The SNP presence may be cultivar-specific and might result in an instability of the G4 structure. This could ultimately result in altered promoter activity.

Some variations may not affect the stability due to the location of the variation site on the structure. For longer G4 motifs (more than four G-stems), proper folding of G4 structures can be sustained by alternative G-stems or by sliding off the G-stems from the variation site to avoid quadruplex instability. Our results showed that four of the remaining SNPs have the potential to interrupt proper folding of their respective G4 structures whose stabilities are dependent on loop length and the site of the G-stems (Supplementary Data 4). Variations that reside in the loop regions or in the G4 flanking regions indicated with gray dots in Fig. 7 have limited effect on the structure.

### Nucleotide-binding-sites (NBS) type disease resistance genes

The most represented group of disease resistance genes are those which code for proteins containing nucleotide-binding-sites (NBS) and leucine-rich repeats (LRR). These NBS domains are known to contain highly conserved cis- and trans- acting elements^73,74^. Defined NBS domains range up to ~ 300 amino acids, which are composed of eight well-known characteristic motifs: P-loop (Kinase-1a), Kinase-2, RNBS-A, RNBS-B, RNBS-C, RNBS-D, GLPL, and MHDV. A thorough investigation was performed for barley putative NBS genes containing G4 motifs using a MEME (multiple expectation maximization for motif elicitation) analysis^75^ to check for the conservation of the NBS domain.

Of 302 NBS genes, 62 contained G4 motifs within the gene or 500 bp flanking regions. The MEME results identified the P-loop, GLPL, Kinase-2, RNBS-B, and MHDV among the 20 motifs that showed the highest levels of similarity between the NBS genes (Supplementary Figure 2). From the MEME results, it was found that not all NBS genes contained all five motifs, with an average of 2–3 of these motifs appearing in each NBS gene. Our results showed a distribution: 16 of the G4 containing NBS genes have all five motifs, 17 genes have 4 motifs, 3 genes have 3 motifs, 2 genes have 2 motifs, and 3 genes have only one motif. Intriguingly, P-loop, Kinase-2, RNBS-B, and GLPL motifs were located in the same order in all barley NBS genes, whereas the MHDV motif was observed either as the first from the N-terminus or C-terminus.

The remaining 21 NBS genes does not contain any of the known NBS gene motifs. Identification of the additional regulatory elements would help in the characterization of these NBS genes without known motifs. Therefore, we examined the distribution of the four different G4 motifs discussed in this study; perfect G4s, imperfect G4s, imperfect G4s with long loops, and multimeric G4s (Table 1). Although only a small subset of the NBS genes contain perfect G4 motifs, 62 out of 302 NBS genes, our results represented striking differences in the G4 motif containing genes. We showed that the NBS genes that does not contain any known NBS motif covered in this study, are highly enriched for the G4 motifs such that multiple occurrences of the same class of the G4 motifs were observed within a gene (Table 1). Intriguingly, G4 density increased from 93 perfect G4 motifs per Mb (Supplementary Table 1) to a 307 perfect G4 motifs per Mb. Another interesting finding is the multimeric G4 motif distribution. Our results showed that the gene, HORVU.MOREX.r2.5HG0444750.1, contains two multimeric G4 motifs in close proximity (within the 500 bp upstream region on the antisense strand, specifically at chr5H:_591,367,833–591,367,879 and chr5H:_591,367,887–591,367,933). As other NBS genes do not contain any G4 motifs within the gene body and 500 bp flanking regions, the drastic change in the localization of the G4 motifs in these 21 NBS genes suggests regulatory functions of these G4 motifs.

**Table 1:** Composition of the four different G4 motifs within the NBS genes.

Chromosome	GeneID	Gene length (bp)	The number of				G4 density (× 10^6^)			
			Perfect G4s	Imperfect G4s (L = 7)	Imperfect G4s (L = 12)	Multimeric G4s	Perfect G4s	Imperfect G4s (L = 7)	Imperfect G4s (L = 12)	Multimeric G4s
Chr1h	HORVU.MOREX.r2.1HG0002610.1	8661	2	13	18	0	231	1501	2078	0
Chr1h	HORVU.MOREX.r2.1HG0002700.1	14,874	3	19	28	0	202	1277	1882	0
Chr1h	HORVU.MOREX.r2.1HG0011680.1	3940	1	11	17	0	254	2792	4315	0
Chr1h	HORVU.MOREX.r2.1HG0005090.1	4255	2	9	14	0	470	2115	3290	0
Chr2h	HORVU.MOREX.r2.2HG0078850.1	8501	3	21	29	0	353	2470	3411	0
Chr2h	HORVU.MOREX.r2.2HG0080510.1	2011	1	5	5	0	497	2486	2486	0
Chr2h	HORVU.MOREX.r2.2HG0090330.1	10,938	3	29	38	0	274	2651	3474	0
Chr2h	HORVU.MOREX.r2.2HG0134150.1	1748	1	9	12	0	572	5149	6865	0
Chr2h	HORVU.MOREX.r2.2HG0163720.1	1519	2	14	12	0	1317	9217	7900	0
Chr3h	HORVU.MOREX.r2.3HG0212040.1	1249	1	3	6	0	801	2402	4804	0
Chr3h	HORVU.MOREX.r2.3HG0260420.1	1700	1	9	9	0	588	5294	5294	0
Chr3h	HORVU.MOREX.r2.3HG0182800.1	11,602	1	24	25	0	86	2069	2155	0
Chr5h	HORVU.MOREX.r2.5HG0399660.1	1480	1	1	2	0	676	676	1351	0
Chr5h	HORVU.MOREX.r2.5HG0444750.1	2077	3	4	7	2	1444	1926	3370	963
Chr5h	HORVU.MOREX.r2.5HG0445390.1	7727	1	6	12	0	129	776	1553	0
Chr6h	HORVU.MOREX.r2.6HG0448770.1	7464	1	18	28	0	134	2412	3751	0
Chr6h	HORVU.MOREX.r2.6HG0456620.1	51,451	8	122	171	0	155	2371	3324	0
Chr6h	HORVU.MOREX.r2.6HG0487860.1	1872	1	4	7	0	534	2137	3739	0
Chr6h	HORVU.MOREX.r2.6HG0519760.1	3227	5	5	5	1	1549	1549	1549	310
Chr6h	HORVU.MOREX.r2.6HG0522990.1	1270	5	2	2	0	3937	1575	1575	0
Chr7h	HORVU.MOREX.r2.7HG0531470.1	5556	1	9	14	0	180	1620	2520	0
Total		153,122	47	337	461	3	307	2201	3011	20

21 NBS genes that lack known NBS motifs but overlap with a perfect G4 motif are represented.

Gene length accounts for the length of the gene and the 500 bp of both upstream and downstream of the gene.

## Conclusions

Accumulated evidence has suggested the formation of stable structures of G-quadruplexes in several phylogenetically distant species. Although the formation of quadruplex structures has been already shown in vitro and in vivo for mostly human genome, regulatory functions of these structures are still in the need of further discovery and verification, especially in plant genomes. Limited information is available for the G-quadruplex structures in plant genomes. Understanding the localization and functional roles of these structures in plants will facilitate the development of plants with better traits.

Here, we performed a genome-wide discovery of putative G4 motifs in the barley genome. Different patterns of G4 motifs could be localized in various other important regulatory sites and could be associated with key molecular functions; however, in this study we mainly focused on the general pattern of {G_3+_L_1−7_}_3+_G_3+_ since it has been supported to form stable G4-structures through in vitro studies in plants and other species^23,24,76^. Our results showed that these G4 motifs are devoid of single-nucleotide polymorphisms and only a limited number of G4 motifs contain polymorphisms that could disrupt proper folding of G4 structures, suggesting a functional role for these structures.

Our results showed the prevalence of G4 motifs around high-confidence barley genes with a similar density distribution across genome and around genes in the wheat genome. Specifically, G4-motifs were enriched around three positions with respect to gene structural elements: one at immediate upstream of the start codon corresponding to the 5′ UTR, the second one at the immediate downstream of the start codon corresponding to the first CDS, and the final one at the start of the intron 1. The enrichment around important regulatory regions implies the functional importance of the G4 motifs in the transcription, translation, and replication. Additionally, we noted that these peak regions could be observed with appropriate selection of the window sizes (< 100 bp) to plot distribution of G4 motif density around genes. Our study here provides evidence that previous genome analyses that used a large window size (> 1000 bp) may have spuriously merged distinct G4 density peaks into one single peak.

Our comparative analyses in human, Arabidopsis, maize, rice and sorghum demonstrate that the G4 distribution around genic regions are relatively similar in the species studied, except in the case of Arabidopsis. Based on the results shown here, the difference in the distribution of G4 structures in Arabidopsis, especially the lack of pronounced G4 peaks, cannot be explained solely by computational smoothing or lack of 5′ UTR annotation data, hinting that the difference may be biological. Specifically, given the enrichment of G4 motifs within the plant retrotransposons^59^, the low number of G4 motifs observed in the Arabidopsis genome may be explained by the repetitive content of the genome, where TEs are less abundant in the Arabidopsis genome^63^.

Several G4 containing genes were shown to be conserved between wheat and barley across the chromosomes. Additionally, we showed that the genes containing G4 motifs are associated with various biological processes, among which the functions like transcription initiation and hydrolase activity appears to be conserved among several monocot species. We provided examples of both perfect and imperfect G4 motifs around important genes like NBS type disease resistance genes. Overall, our findings imply functional importance of G4 motifs and indicate both the sequence and functional conservation among monocot species. We believe that the determination of the distribution of G4 motifs can help elucidate genomic hotspots with biological significance, thus revealing their functional importance in gene regulation.

## Methods

### Whole genome sequence assemblies and annotations

The TRITEX genome assembly of barley cultivar Morex^26^ was obtained from e!DAL PGP repository^77^ together with high-confidence annotation data (v2). Any unassigned scaffolds which were merged to form unmapped pseudomolecules (chrUn), and the relevant genes mapped to them, were disregarded for the purpose of the analysis. SNPs for barley were retrieved from the Barley 50k iSelect SNP array^71^.

The wheat reference genome assembly (IWGSC Chinese Spring RefSeq v1.0) was obtained from the wheat-URGI database together with high-confidence gene annotation data (v1.1)^25^. For comparison purposes, five genomes were analyzed, including *Homo sapiens*, *Arabidopsis thaliana*, *Zea mays*, *Oryza sativa japonica,* and *Sorghum bicolor*. The whole genome sequence and annotations for *Oryza sativa japonica* was obtained from TIGR version 7^78^. For all other species, the genome assembly sequences and annotations were retrieved from the NCBI genome database, including human (*Homo sapiens*—GCF_000001405.39), Arabidopsis (*Arabidopsis thaliana—GCF_000001735.4*)*,* maize (*Zea mays—GCF_000005005.2, B73 RefGen_v4 and* GCF_902167145.1*, B73 RefGen_v5*) (*also available* at the Maize Genetics and Genomics Database (MaizeGDB—https://www.maizegdb.org)^79^ and sorghum (*Sorghum bicolor—GCF_000003195.3*)^80^.

### In silico identification of G4 motifs

Canonical G4 motifs are generally defined as at least four runs of G-stems separated by stretches of loops. Previous in vitro evidence supported the canonical (G_3+_L_1−7_)_3+_G_3+_ pattern as a G4 motif which forms stable G4 structures^76^. Therefore, we identified the canonical G4 motifs (also referred as G4 motifs or perfect G4 motifs throughout the manuscript) with the (G_3+_L_1−7_)_3+_G_3+_ pattern across the barley Morex v2 genome, using a custom python script (https://github.com/dariober/bioinformatics-cafe/tree/master/fastaRegexFinder), which is a similar implementation of the quadparser algorithm^24^. The (G_3+_L_1−7_)_3+_G_3+_ pattern is formed by at least three consecutive G bases (G-stem) and a loop between G-stems with a length of 1–7 bases. Similar to previous studies^33^, we restricted classification of G4 motifs to a minimum of four G-stems with nonoverlapping motifs, such that any continuous stretch of G4 motif counted just once. Both sense and antisense strands of the G4 motifs were screened. For the identification of the multimeric G4s, we searched for the G4 motifs with at least 8 G-stems (‘(G_3+_L_1-7_)_7+_G_3+_’).

Imperfect G4 motifs containing one bulges of short length (up to 2 bases) were identified by the QPARSE algorithm^46^. G-stem length was limited to 3 and 4 bases. Two patterns were searched for regular loop size of 7 bases (-m 3 -M 4 -L 7 -g1 -l2) and long loop size of 12 bases (-m 3 -M 4 -L 12 -g1 -l2). To identify G4 motifs on the antisense strand, the same runs were performed to search for C bases (-b C). All QPARSE outputs were processed further by QPARSE_parser to merge overlapping motifs into one motif (-m) and to convert the results into GFF file format (-g). Motifs identified on the sense and on the antisense strands were saved as a single file after QPARSE_parser.

### G4 motif distribution analysis

Total gene length and CDS regions were extracted from the annotation files which were downloaded from publicly available databases. From the gene annotations, 10,000 bp regions centered at CDS start sites were retrieved. The total number of bases overlapping with a G4 motif were calculated for each base position relative to the CDS start sites. For comparison purposes, the total number of bases were averaged by the total number of CDSs for each species separately. G4 density was defined as the total number of G4 motifs over a region, and G4 frequency was defined as the number of bases overlapped at a region.

Observed G4 density at genic regions was compared against the expected G4 density for the whole genome using random shuffling of the high-confidence genes across the genome. High-confidence gene locations were permuted among the genome using bedtools^81^ shuffle function. Random sequences were created by genome-wide shuffling and the overlap with the G4 motifs was assessed. Enrichment of the G4 motifs over genic regions was calculated by the observed overlap with the G4 motifs divided by the overlap of the randomly shuffled sequences with the G4 motifs.

A random dataset was created for a comparison within gene structural regions, corresponding to CDS, intron, 1000 bp (1k) upstream, and 1k downstream of a gene, using the python random module. We created three random datasets, using the python random module, with equal nucleotide probabilities and calculated G4 density within each random dataset. The G4 density was similar among the random datasets so we kept the random dataset with the median G4 density.

### Circos plot and dataset generation

For the synteny analysis, the barley and wheat proteins were compared against each other using BLASTX^82^ with stringent filter criteria of a minimum of 30 amino acids as alignment length and a minimum of 75% sequence similarity. Orthologous genes were identified by a reciprocal best hit approach whereby the best hit for barley was also the best hit for wheat.

Transposable element annotations were downloaded for IWGSC wheat RefSeq v1.0 from the wheat-URGI database https://urgi.versailles.inra.fr/download/iwgsc/IWGSC_RefSeq_Annotations/v1.0/) and for the barley cv. Morex v2 from the Plant Genomics and Phenomics Research Data Repository (https://doi.org/10.5447/IPK/2019/8). Bedtools^81^ was used to make windows within a genome and to create the histogram data by the overlapping features within each window. Circos^54^ was used to draw circular visualization of wheat and barley chromosomes and gene features. Input files used in the generation of the Circos plot provided in the Supplementary Data 3.

### Functional analysis of G4 containing genes

Gene Ontology (GO) annotations for high-confidence gene models were extracted from GrainGenes^28^ genome tracks for high-confidence (HC) barley genes. GO enrichment of genes was conducted using the BiNGO plugin for the Cytoscape (v3.7.2) visualization tool^67^ and default statistical parameters were used for the hypergeometrical statistical test, along with a Benjamini and Hochberg false discovery rate (FDR) correction (significance level 0.05). Comparison of two or more enrichment results were compared using WEGO v2 web tool^83^.

## Supplementary Information

Supplementary Information 1.

Supplementary Information 2.

Supplementary Information 3.

Supplementary Information 4.

Supplementary Information 5.

## Data Availability

The G4 motif sequences are publicly available at GrainGenes^28^. Supplementary Data 1 contains a bed file for the G4 motifs identified in the barley cv. Morex v2 reference genome. Supplementary Data 2 contains functional annotations and GO term enrichments for genes containing G4 motifs within the gene body and 500 bp flanking regions of high-confidence gene models. Supplementary Data 3 contains the input files that were used in the generation of the Circos plot. Supplementary Data 4 contains the list of SNPs located within the barley G4 motifs.
