# Supplementary figures and images for: Genome-wide discovery of G-quadruplexes in barley
Source: Sci Rep. 2021 Apr 12;11:7876. doi: 10.1038/s41598-021-86838-3 (PMC8041835; doi:10.1038/s41598-021-86838-3)

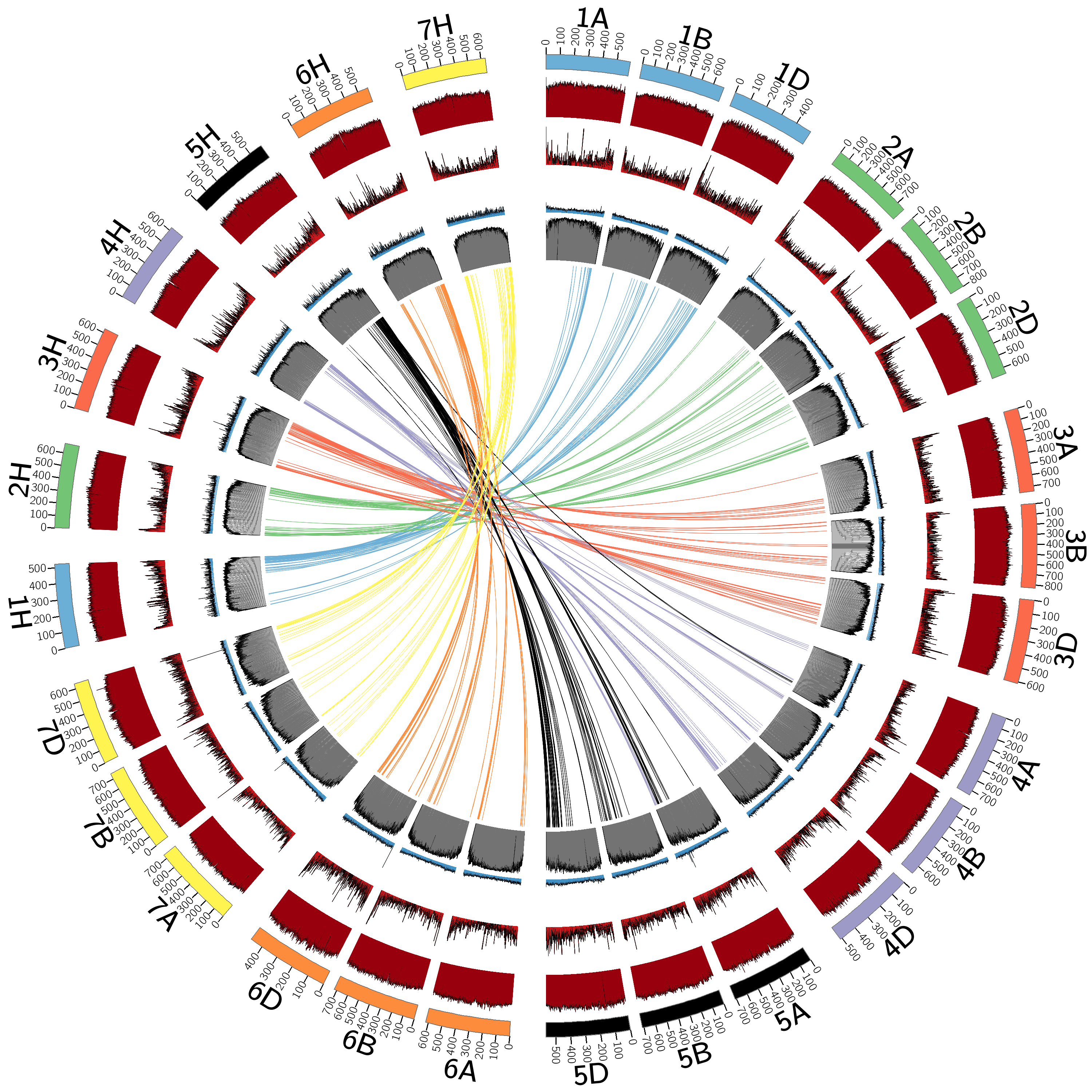

Supplement: Supplementary file 2 — Supplementary Information 2. [file 41598_2021_86838_MOESM2_ESM.zip › circos/circos.png]
