# Supplementary material for: Genome-wide discovery of G-quadruplexes in barley
Source: Sci Rep. 2021 Apr 12;11:7876. doi: 10.1038/s41598-021-86838-3 (PMC8041835; doi:10.1038/s41598-021-86838-3)
Supplement: Supplementary file 5 — Supplementary Information 5. [file 41598_2021_86838_MOESM5_ESM.docx]

**Supplementary Information**

**Genome-wide discovery of G-quadruplexes in barley**

H. Busra Cagirici^1^, Hikmet Budak^2,3^, Taner Z. Sen^1*^

^1^U.S. Department of Agriculture - Agricultural Research Service, Western Regional Research Center, Crop Improvement Genetics Research Unit, 800 Buchanan St., Albany, CA 94710 USA

^2^Montana BioAg Inc., MT, USA

^3^Agrogen, LLC., NE, USA

^*^To whom correspondence should be addressed. E-mail: [taner.sen@usda.gov](mailto:taner.sen@usda.gov)

**List of Supplementary Information**

**Supplementary** **Data 1** – G4 motifs as BED file

**Supplementary** **Data 2** – Functional annotation and GO term enrichment for the genes containing G4s

**Supplementary Data 3** – Input files for Circos

**Supplementary** **Data 4** – SNPs located within the G4 motifs

**Supplementary Figure 1.** G4 frequency across 10,000 bp centered at the start of the first CDS on the whole genome and on each chromosome. G4 frequency was calculated as total number of G4 motifs per Mb at each position averaged by a window size of 100 bp. Blue lines indicate G4 motifs on sense strands, green lines indicate G4 motifs on the antisense strands, and red lines indicate G4 motifs on either sense or antisense strands combined.

**Supplementary Figure 2.** MEME motifs aligned to NBS genes. Annotation of motif sequences in terms of NBS related motifs were shown below. Short list of NBS genes and motif locations is shown here.

**Supplementary Table 1**. Distribution of predicted G4 motifs (G4s) in the barley genome and its comparison to other species. Genic regions include regions annotated as HC genes; CDS and introns. G4 density indicates total number of G4 motifs per bp. Chromosomes which possess the highest and lowest G4 densities were highlighted with italic bold letters. Barley genome is the TRITEX genome assembly of barley cultivar Morex v2^1^ and the wheat genome is the IWGSC RefSeq v1.0 genome assembly of the wheat cultivar Chinese Spring^2^.

**Supplementary Table 2**. Composition of the TE superfamilies in the G4 motifs

**Supplementary Table 3.** A list of G4 containing barley genes which were assigned to response to cold function. Homoeologous wheat genes were also shown.

**Supplementary Table 4**. Enriched GO terms for the genes containing G4 motifs within peak1. GO terms are shown if more than 50% of the genes associated with the GO terms contain a G4 motif within peak1.

**Supplementary Figures**


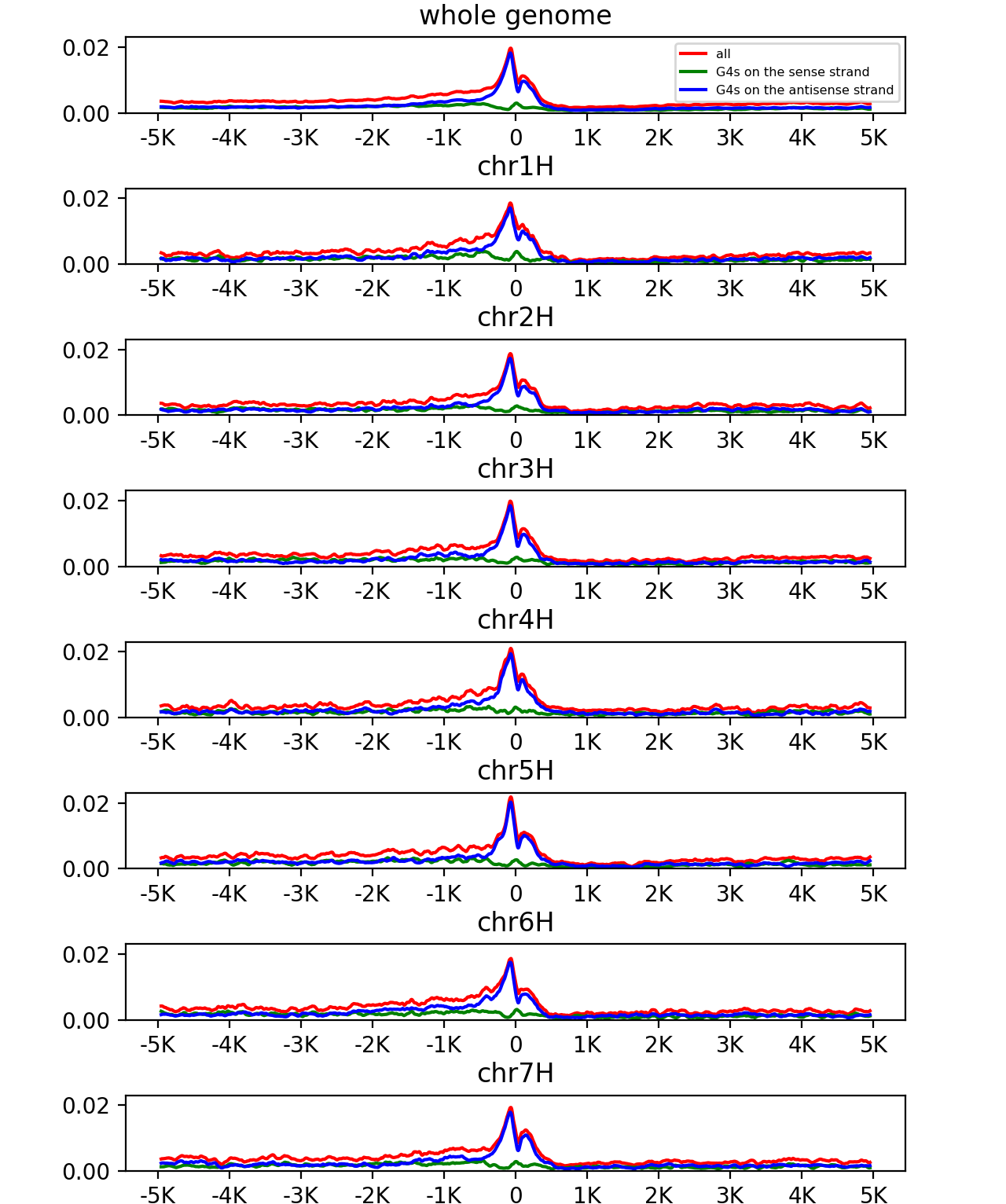
**Supplementary Figure 1.** G4 frequency across 10,000 bp centered at the start of the first CDS on the whole genome and on each chromosome. G4 frequency was calculated as total number of G4 motifs per Mb at each position averaged by a window size of 100 bp. Blue lines indicate G4 motifs on sense strands, green lines indicate G4 motifs on the antisense strands, and red lines indicate G4 motifs on either sense or antisense strands combined.


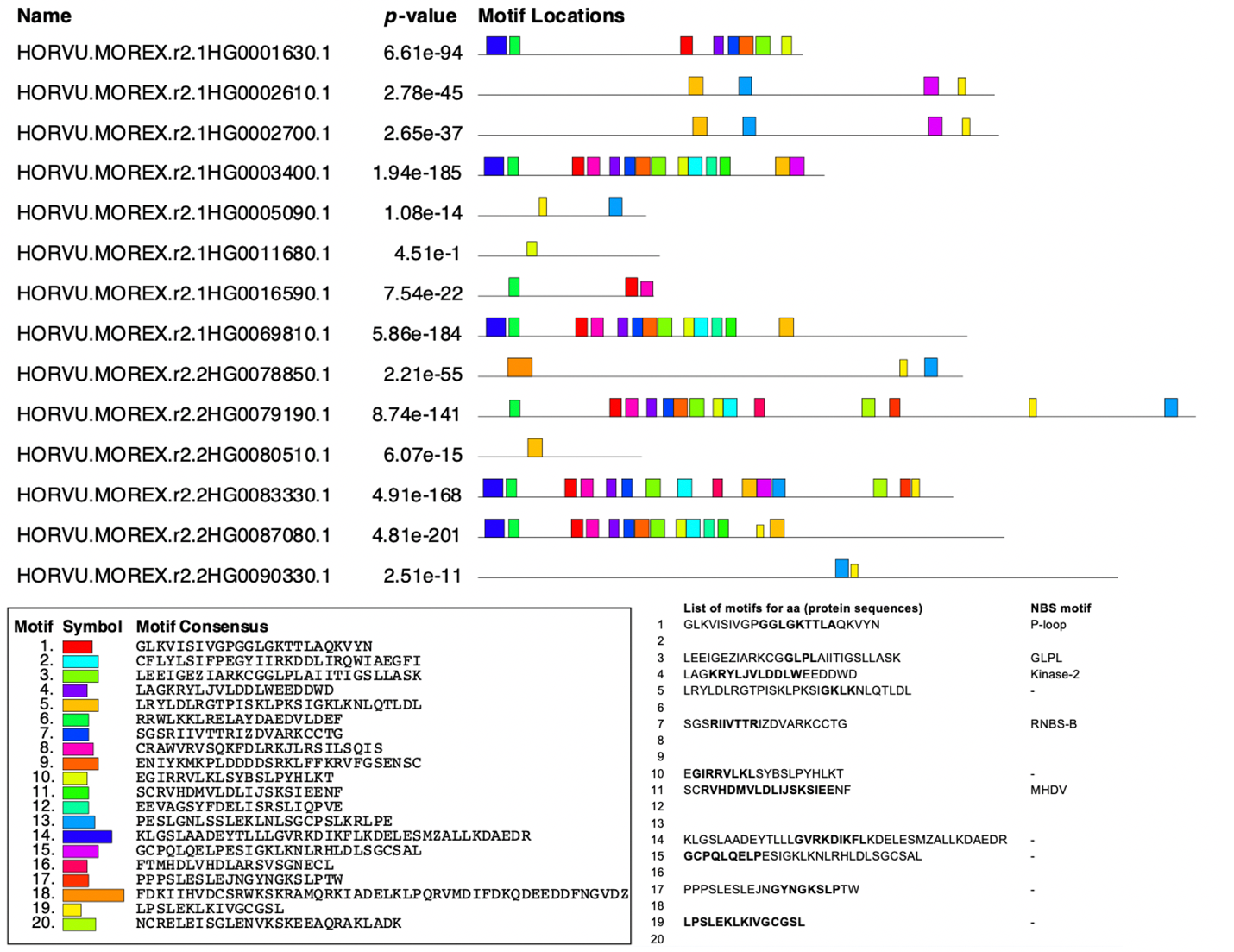


**Supplementary Figure 2.** MEME motifs aligned to NBS genes. Annotation of motif sequences in terms of NBS related motifs were shown below. Short list of NBS genes and motif locations is shown here.

**Supplementary Tables**

**Supplementary Table 1**. Distribution of predicted G4 motifs (G4s) in the barley genome and its comparison to other species. Genic regions include regions annotated as HC genes; CDS and introns. G4 density indicates total number of G4 motifs per bp. Chromosomes which possess the highest and lowest G4 densities were highlighted with italic bold letters. Barley genome is the TRITEX genome assembly of barley cultivar Morex v2^1^ and the wheat genome is the IWGSC RefSeq v1.0 genome assembly of the wheat cultivar Chinese Spring^2^.

|  |  |  | **Whole genome** | | **Genic regions** | |
| --- | --- | --- | --- | --- | --- | --- |
| **Chromosome** | **Chromosome length** | **Average size of G4 motifs** | **# of G4 motifs** | **G4 density**  **(x10^6^)** | **# of G4 motifs** | **G4 density (x10^6^)** |
| **Barley genome** | 4,257,712,555 | 30.4 | 329,098 | 77 | 9,360 | 93 |
| **chr1H** | 522,466,905 | 30.8 | 39,938 | 76 | 1,128 | 90 |
| **chr2H** | 675,310,294 | 30.5 | 52,090 | 77 | 1,499 | 88 |
| **chr3H** | 628,753,756 | 30.2 | 48,096 | 76 | 1,423 | 90 |
| **chr4H** | 624,247,919 | 30.2 | 48,667 | 78 | 1,194 | 93 |
| **chr5H** | 599,018,945 | 30.8 | 46,169 | 77 | 1,484 | 95 |
| ***chr6H*** | ***573,247,234*** | ***30.4*** | ***44,483*** | ***78*** | ***1,080*** | ***87*** |
| ***chr7H*** | ***634,667,502*** | ***30.0*** | ***49,655*** | ***78*** | ***1,552*** | ***104*** |
|  |  |  |  |  |  |  |
| **Wheat genome** | 14,066,280,851 | 27.8 | 1,071,813 | 76 | 34,370 | 93 |
| **Maize genome**  **(v5)** | 2,131,846,805 | 31.2 | 196,521 | **92** | 17,182 | **101** |
| **Maize genome**  **(v4)** | 2,106,338,117 | 31.2 | 190,575 | **90** | 17,267 | **96** |
| **Rice genome** | 382,150,945 | 27.7 | 40,466 | **106** | 8,408 | **85** |
| **Arabidopsis genome** | 119,146,348 | 27.6 | 1,219 | **10** | 346 | **5** |

1. Monat, C. *et al.* TRITEX: Chromosome-scale sequence assembly of Triticeae genomes with open-source tools. *Genome Biology*. doi: 10.1186/s13059-019-1899-5 (2019).

2. Appels, R. *et al.* Shifting the limits in wheat research and breeding using a fully annotated reference genome. *Science*. **361** (6403), doi: 10.1126/science.aar7191 (2018).

**Supplementary Table 2**. Composition of the TE superfamilies in the G4 motifs

| **Repeat class** | **Repeat superfamily** | **Superfamily ID** | **Percentage** |
| --- | --- | --- | --- |
| Class I LTR retrotransposons | | | 85.463 |
|  | Gypsy | RLG | 22.645 |
|  | Copia | RLC | 20.108 |
|  | Unclassified LTR-RT | RLX | 42.373 |
|  | LINE | RIX | 0.316 |
|  | SINE | RSX | 0.022 |
|  |  |  |  |
| Class 2 DNA transposons | |  | 13.872 |
|  | CACTA | DTC | 13.201 |
|  | Mutator | DTM | 0.137 |
|  | Unclassified with TIRs | DTX | 0.012 |
|  | Harbinger | DTH | 0.301 |
|  | Mariner | DTT | 0.004 |
|  | Unclassified class 2 | DXX | 0.180 |
|  | hAT | DTA | 0.002 |
|  | Helitrons | DHH | 0.035 |
|  |  |  |  |
| Unclassified repeats | XXX |  | 0.665 |

**Supplementary Table 3.** A list of G4 containing barley genes which were assigned to response to cold function. Homoeologous wheat genes were also shown.

| **Wheat Homeologs** | **Barley Homeologs** |
| --- | --- |
| TraesCS1A02G091400.1 | HORVU.MOREX.r2.1HG0017850.1 |
| TraesCS1A02G131300.1 | HORVU.MOREX.r2.1HG0027460.1 |
| TraesCS1D02G133000.1 | HORVU.MOREX.r2.1HG0027100.1 |
| TraesCS1B02G293300.1 | HORVU.MOREX.r2.1HG0059440.1 |
| TraesCS1D02G314800.1 | HORVU.MOREX.r2.1HG0055100.1 |
| TraesCS2A02G102200.2 | HORVU.MOREX.r2.2HG0091530.1 |
| TraesCS2A02G456100.1 | HORVU.MOREX.r2.2HG0162740.1 |
| TraesCS2B02G170700.1 | HORVU.MOREX.r2.2HG0098540.1 |
| TraesCS2A02G166600.1 | HORVU.MOREX.r2.2HG0102730.1 |
| TraesCS2D02G102800.1 | HORVU.MOREX.r2.2HG0091620.1 |
| TraesCS2B02G363700.1 | HORVU.MOREX.r2.2HG0146330.1 |
| TraesCS2B02G179900.1 | HORVU.MOREX.r2.2HG0100520.1 |
| TraesCS2B02G458500.1 | HORVU.MOREX.r2.2HG0160460.1 |
|  | HORVU.MOREX.r2.2HG0154810.1 |
| TraesCS3B02G391900.1 | HORVU.MOREX.r2.3HG0252510.1 |
| TraesCS3D02G356500.1 | HORVU.MOREX.r2.3HG0252030.1 |
| TraesCS3A02G264800.1 | HORVU.MOREX.r2.3HG0232790.1 |
| TraesCS3B02G021600.2 | HORVU.MOREX.r2.3HG0184170.1 |
| TraesCS4D02G296400.1 | HORVU.MOREX.r2.4HG0337430.1 |
| TraesCS4D02G144400.2 | HORVU.MOREX.r2.4HG0302020.1 |
| TraesCS4D02G133500.1 | HORVU.MOREX.r2.4HG0295360.1 |
| TraesCS5B02G201700.2 | HORVU.MOREX.r2.5HG0396660.1 |
| TraesCS6B02G371000.2 | HORVU.MOREX.r2.6HG0514950.1 |
| TraesCS6D02G122800.1 | HORVU.MOREX.r2.6HG0468290.1 |
|  | HORVU.MOREX.r2.6HG0500180.1 |
| TraesCS6A02G358500.1 | HORVU.MOREX.r2.6HG0518020.1 |
| TraesCS7A02G490200.1 | HORVU.MOREX.r2.7HG0613230.1 |
| TraesCS7A02G298800.2 | HORVU.MOREX.r2.7HG0579590.1 |
| TraesCS7A02G111300.1 | HORVU.MOREX.r2.7HG0541760.1 |

**Supplementary Table 4**. Enriched GO terms for the genes containing G4 motifs within peak1. GO terms are shown if more than 50% of the genes associated with the GO terms contain a G4 motif within peak1.

| **GO category** | **GO ID** | **Peak1** | **All** | **Description** | **%** |
| --- | --- | --- | --- | --- | --- |
| BP | GO:0008486 | 2 | 2 | diphosphoinositol-polyphosphate diphosphatase activity | 100 |
| BP | GO:0050072 | 2 | 2 | m7G(5')pppN diphosphatase activity | 100 |
| BP | GO:0034431 | 2 | 2 | bis(5'-adenosyl)-hexaphosphatase activity | 100 |
| BP | GO:0034432 | 2 | 2 | bis(5'-adenosyl)-pentaphosphatase activity | 100 |
| BP | GO:0071543 | 2 | 2 | diphosphoinositol polyphosphate metabolic process | 100 |
| BP | GO:0018800 | 2 | 2 | 5-oxopent-3-ene-1,2,5-tricarboxylate decarboxylase activity | 100 |
| CC | GO:0000786 | 3 | 5 | nucleosome | 60 |
